# Chest Wall Reconstruction in Ewing Sarcoma Using a Radioprotective Spacer

**DOI:** 10.70352/scrj.cr.25-0433

**Published:** 2025-10-08

**Authors:** Hiroko Yoshizawa, Keita Terui, Mitsuyuki Nakata, Ayako Takenouchi, Shugo Komatsu, Satoru Oita, Yunosuke Kawaguchi, Katsuhiro Nishimura, Wataru Kudo, Genta Ozeki, Moeko Hino, Tomoko Okunushi, Yoshiharu Yamashita, Rintaro Harada, Shinya Hattori, Tomoro Hishiki

**Affiliations:** 1 Department of Pediatric Surgery, Chiba University Graduate School of Medicine, Chiba, Chiba, Japan; 2 Department of Pediatrics, Chiba University Graduate School of Medicine, Chiba, Chiba, Japan; 3 Department of Radiology, Chiba University Hospital, Chiba, Chiba, Japan

**Keywords:** Ewing sarcoma, radioprotective spacer, chest wall

## Abstract

**INTRODUCTION:**

Ewing sarcoma is a rare malignant tumor that primarily affects children and adolescents, and approximately 20% occur in the chest wall, where achieving local control is challenging due to the need for extensive resection and the proximity to vital organs. Adjuvant radiotherapy improves outcomes but carries a risk of severe complications such as radiation pneumonitis. Radioprotective spacers have been used for abdominal and pelvic tumors, but their use in chest wall tumors is rarely reported. We report a chest wall Ewing sarcoma case in which a radioprotective spacer using expanded polytetrafluoroethylene and a water-inflatable expander was placed to reduce radiation-related organ damage.

**CASE PRESENTATION:**

A 15-year-old female presented with a large Ewing sarcoma arising from the left chest wall, measuring 121 × 166 × 93 mm. After diagnosis by wedge biopsy and confirmation of *EWSR1::FLI1* fusion, she received neoadjuvant chemotherapy based on the JESS04 protocol. Following significant tumor shrinkage, surgical resection was performed via a 20‑cm thoracotomy. En bloc removal included the tumor, the 5th and 6th ribs, and a portion of the adjacent lung. To reconstruct the chest wall and protect adjacent organs from high‑dose postoperative radiotherapy, a novel radioprotective spacer was inserted. The spacer was constructed by sandwiching a water‑inflatable tissue expander between two expanded polytetrafluoroethylene sheets. This design provided dual functionality: it reinforced the large chest wall defect and physically distanced the left lung from the radiation field. The expander was filled with 70 mL of water to create an adjustable spacer volume and could be deflated if postoperative symptoms occurred. The patient underwent intensity‑modulated radiation therapy with a total dose of 50.4 Gy without respiratory complications. After radiotherapy, the expander was removed, while the expanded polytetrafluoroethylene sheets remained in place to maintain chest wall integrity. At 4 years postoperatively, the patient remains disease‑free and symptom‑free.

**CONCLUSIONS:**

This case illustrates the feasibility and safety of combining a radioprotective spacer with structural chest wall reconstruction in pediatric sarcoma. The dual‑purpose design may offer an effective strategy for minimizing radiation‑related toxicity in thoracic tumors requiring multimodal treatment.

## Abbreviations

ePTFE
: expanded polytetrafluoroethylene
IMRT
: intensity-modulated radiation therapy
QUANTEC
: Quantitative Analysis of Normal Tissue Effects in the Clinic

## INTRODUCTION

Ewing sarcoma is a rare malignant tumor that predominantly affects children, adolescents, and young adults. It typically arises in bones or soft tissues and is the second most common primary bone cancer in the pediatric population after osteosarcoma.^1)^ Approximately 20% of Ewing sarcomas occur in the chest wall,^2)^ where achieving local control presents unique challenges. Surgical resection often requires the removal of a large portion of the chest wall to obtain adequate margins, sometimes necessitating reconstruction.

Radiotherapy combined with surgery plays a crucial role in improving outcomes. However, high-dose radiation—essential for tumor control—poses significant risks to surrounding organs, especially the lungs. Radiation pneumonitis is a serious and potentially life-threatening complication.

Radioprotective spacers have increasingly been used to distance radiosensitive organs from irradiation fields in pediatric and adult patients, particularly for pelvic or abdominal tumors.^3,4)^ However, reports of their use for chest wall tumors remain limited. Here, we present a case of a large chest wall Ewing sarcoma treated with wide resection and adjuvant radiotherapy, where a radioprotective spacer was placed using a novel technique combining expanded polytetrafluoroethylene (ePTFE) sheets and a water-inflatable tissue expander.

## CASE PRESENTATION

**Fig. 1:**
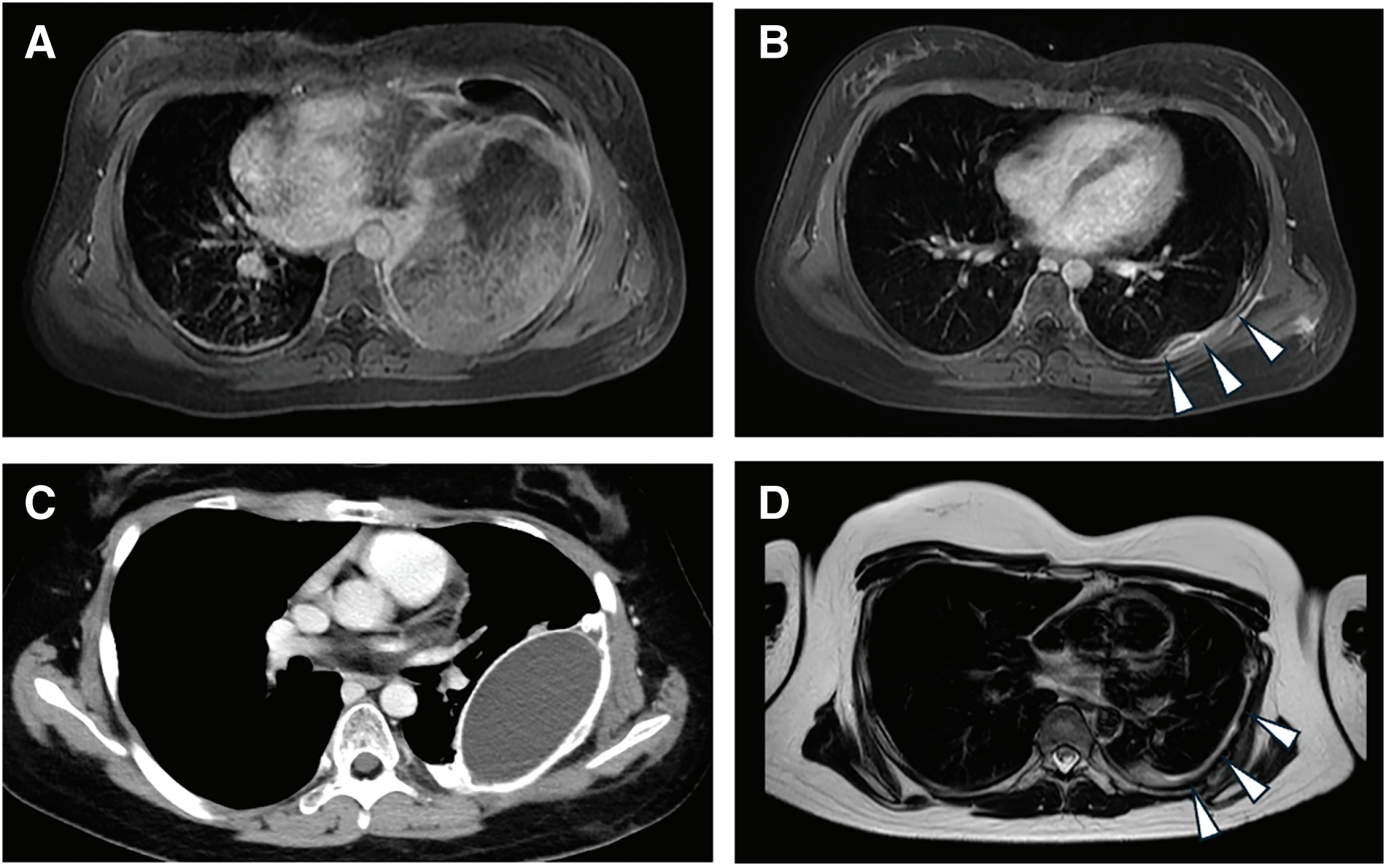
Tumor and tumor bed imaging findings (axial sections) over time. (**A**) MRI at diagnosis (contrast-enhanced fat-suppressed T1-weighted image). (**B**) MRI before definitive surgery (contrast-enhanced fat-suppressed T1-weighted image). White arrowheads indicate the residual tumor. (**C**) Contrast-enhanced CT after placement of the radioprotective spacer. The tissue expander–ePTFE sheet complex is depicted as an oval structure. (**D**) MRI 4 years after completion of treatment (T2-weighted image). White arrowheads indicate the bilayer ePTFE sheet. ePTFE, expanded polytetrafluoroethylene

A previously healthy 15-year-old female presented with a 2-month history of left-sided chest pain. MRI revealed a well-encapsulated solid mass measuring 121 × 166 × 93 mm in the left upper hemithorax, originating from the 5th intercostal space (**Fig. 1A**). Moderate pleural effusion was noted, but cytological examination was negative for malignancy. Wedge biopsy via mini-thoracotomy confirmed Ewing sarcoma (*EWSR1::FLI1* fusion gene positive).

Neoadjuvant chemotherapy with vincristine, doxorubicin, cyclophosphamide, ifosfamide, and etoposide was administered per the JESS04 protocol (VDC-IE regimen).^5)^ MRI after 12 weeks showed marked tumor reduction (**Fig. 1B**), and surgical resection was performed.

**Fig. 2:**
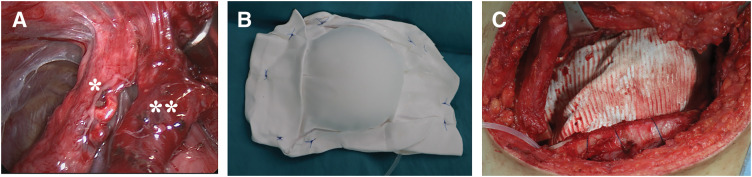
Intraoperative findings. (**A**) Tumor appearance at surgery. (**B**) Tissue expander–ePTFE sheet complex forming the radioprotective spacer. (**C**) Spacer applied to the tumor bed. *, Lung; **, tumor

A 20-cm thoracotomy was made along the 5th intercostal space. The tumor primarily involved the 5th intercostal space and adhered to adjacent lung tissue (**Fig. 2A**). The intraoperative frozen section revealed residual viable tumor. The tumor was resected en bloc, along with the 5th and 6th ribs and part of the right upper lobe. To ensure radicality, the lung parenchyma attached to the tumor was resected with a 2-cm margin.

To reinforce the chest wall defect and simultaneously function as a spacer, 2 ePTFE sheets (GORE DUALMESH; W. L. Gore & Associates, Flagstaff, AZ, USA) were customized and sutured together with their corduroy side facing outwards to sandwich a PMT tissue expander (round type; 100 × 100 mm) (PMT Corporation, Chanhassen, MN, USA) (**Fig. 2B**). The expander was filled with 70 mL of water to adjust spacer thickness and fixed to the chest wall (**Fig. 2C**). The surgical margin was negative on pathological assessment.

Postoperative recovery was uneventful, and the patient experienced no respiratory compromise. CT confirmed proper spacer positioning (**Fig. 1C**). The patient subsequently received IMRT with 50.4 Gy in 28 fractions targeting the surgical bed while sparing the lung compartment separated by the spacer (**Supplementary Fig. 1**). After completing radiotherapy, the PMT tissue expander was removed under general anesthesia, leaving the ePTFE sheets for continued reinforcement (**Fig. 1D**). The patient remains disease-free and symptom-free 4 years postoperatively.

## DISCUSSION

This case demonstrates the use of a radioprotective spacer in managing chest wall Ewing sarcoma. While spacers have been well-documented in abdominal and pelvic tumors to protect adjacent organs from high-dose radiation, reports of their use in thoracic tumors remain rare.^3,4,6,7)^ This case illustrates that a spacer can be safely and effectively used in the thoracic cavity to reduce lung radiation exposure while simultaneously reinforcing the chest wall.

Radiotherapy is essential for local control in Ewing sarcoma, particularly when complete resection is difficult.^8,9)^ However, required doses—typically over 40–50 Gy—pose significant risks to surrounding thoracic organs. The lungs are especially vulnerable, and radiation-induced pneumonitis or fibrosis can lead to severe morbidity, particularly in young patients with long life expectancy.^10)^ Thus, reducing incidental lung dose is critical.

In this case, IMRT was used to optimize dose distribution. Nonetheless, a novel spacer constructed by sandwiching a water-inflatable tissue expander between two ePTFE sheets further minimized pulmonary exposure. In thoracic radiotherapy, the percentage of lung volume receiving ≥20 Gy (V20) is generally used as the standard predictor of symptomatic radiation pneumonitis. According to the QUANTEC (Quantitative Analyses of Normal Tissue Effects in the Clinic) lung report,^11)^ it is prudent to keep V20 ≤30%–35% and mean lung dose ≤20–23 Gy to limit the risk of pneumonitis to approximately 20%. In the present case, a dosimetric simulation suggested that spacer placement reduced the lung V20 from 35.1% to 28.3% and the mean lung dose to 13.9 Gy, indicating that the spacer likely contributed to lowering this risk.

This spacer design offered several advantages. First, the expander’s thickness could be adjusted by filling it with water, allowing precise control of the distance between the radiation target and adjacent lung tissue. In the unlikely event of postoperative respiratory symptoms due to lung compression, the expander could be easily deflated to relieve symptoms, offering an additional safety margin.

Second, only the expander was removed after radiotherapy, while the ePTFE sheets were left in place as permanent reinforcement. Large chest wall defects often require synthetic materials like ePTFE to prevent complications such as flail chest.^12)^ Our technique—wrapping the expander with ePTFE sheets and securing them to the chest wall—ensured both spacer stability during irradiation and durable structural support afterward. Since water-inflatable expanders cannot be directly sutured to tissue, this method provided secure placement and a seamless transition from temporary spacer to long-term reinforcement.

This dual-purpose strategy of combining a spacer with structural reinforcement in a single device may offer a valuable option for cases requiring both high-dose radiotherapy and extensive chest wall resection. It also suggests potential for further innovation in spacer design tailored to thoracic oncologic surgery.

## CONCLUSIONS

In conclusion, this case highlights the potential of incorporating a radioprotective spacer in the management of chest wall Ewing sarcoma. The adjustable water-filled tissue expander sandwiched between ePTFE sheets provided effective lung protection during radiotherapy while simultaneously contributing to chest wall reconstruction. While further studies and clinical experience are needed to evaluate the broader applicability of this technique, we believe it represents a promising advance in multidisciplinary care for thoracic malignancies.

## SUPPLEMENTARY MATERIAL

Supplementary Figure 1 IMRT dose distribution displayed on planning CT in axial, coronal, and sagittal planes. The oval light-blue marked structure indicates the spacer.

## DECLARATIONS

### Availability of data and materials

The dataset supporting the conclusions of this article is included within the article.

## References

[1] Saenz NC, Hass DJ, Meyers P, et al. Pediatric chest wall Ewing’s sarcoma. J Pediatr Surg 2000; 35: 550–5. 10770379 10.1053/jpsu.2000.0350550

[2] Worch J, Ranft A, DuBois SG, et al. Age dependency of primary tumor sites and metastases in patients with Ewing sarcoma. Pediatr Blood Cancer 2018; 65: e27251. 29856530 10.1002/pbc.27251

[3] Tang Q, Zhao F, Yu X, et al. The role of radioprotective spacers in clinical practice: a review. Quant Imaging Med Surg 2018; 8: 514–24. 30050786 10.21037/qims.2018.06.06 PMC6037953

[4] Kimura M, Asai K, Iwata H, et al. Impact on dose distribution and volume changes of a bioabsorbable polyglycolic acid spacer during chemo-proton therapy for a pediatric Ewing sarcoma. J Radiat Res 2020; 61: 952–8. 32960269 10.1093/jrr/rraa087 PMC7674708

[5] Chin M, Yokoyama R, Sumi M, et al. Multimodal treatment including standard chemotherapy with vincristine, doxorubicin, cyclophosphamide, ifosfamide, and etoposide for the Ewing sarcoma family of tumors in Japan: Results of the Japan Ewing Sarcoma Study 04. Pediatr Blood Cancer 2020; 67: e28194. 32077253 10.1002/pbc.28194

[6] Arceo-Olaiz R, Smith EA, Stokes C, et al. Use of perirectal hyaluronic acid spacer prior to radiotherapy in a pediatric patient with bladder rhabdomyosarcoma: a case report. Urology 2023; 181: 136–40. 37453583 10.1016/j.urology.2023.06.018

[7] Ahmad F, Suominen JS, Hassan Z, et al. Use of a tissue expander as a radio-protective spacer with a latissimus dorsi flap in the management of a peripheral primitive neuroectodermal tumour (pPNET). J Plast Reconstr Aesthet Surg 2013; 66: e169–71. 23582507 10.1016/j.bjps.2013.02.022

[8] Andreou D, Ranft A, Gosheger G, et al. Which factors are associated with local control and survival of patients with localized pelvic Ewing’s sarcoma? A retrospective analysis of data from the EURO-EWING99 trial. Clin Orthop Relat Res 2020; 478: 290–302. 31580267 10.1097/CORR.0000000000000962 PMC7438129

[9] Bedetti B, Wiebe K, Ranft A, et al. Local control in Ewing sarcoma of the chest wall: results of the EURO-EWING99 trial. Ann Surg Oncol 2015; 22: 2853–9. 26104542 10.1245/s10434-015-4630-0

[10] Armenian SH, Landier W, Francisco L, et al. Long-term pulmonary function in survivors of childhood cancer. J Clin Oncol 2015; 33: 1592–600. 25847925 10.1200/JCO.2014.59.8318 PMC4417729

[11] Marks LB, Bentzen SM, Deasy JO, et al. Radiation dose-volume effects in the lung. Int J Radiat Oncol Biol Phys 2010; 76 (Suppl): S70–6. 20171521 10.1016/j.ijrobp.2009.06.091 PMC3576042

[12] Sarcon AK, Selim OA, Mullen BL, et al. Expanded polytetrafluoroethylene mesh in chest-wall reconstruction: a 27-year experience. J Thorac Cardiovasc Surg 2025; 169: 303–13.e2. 38879120 10.1016/j.jtcvs.2024.05.026

