# Supplementary figures and images for: Chest Wall Reconstruction in Ewing Sarcoma Using a Radioprotective Spacer
Source: Surg Case Rep. 2025 Oct 8;11(1):25-0433. doi: 10.70352/scrj.cr.25-0433 (PMC12511784; doi:10.70352/scrj.cr.25-0433)

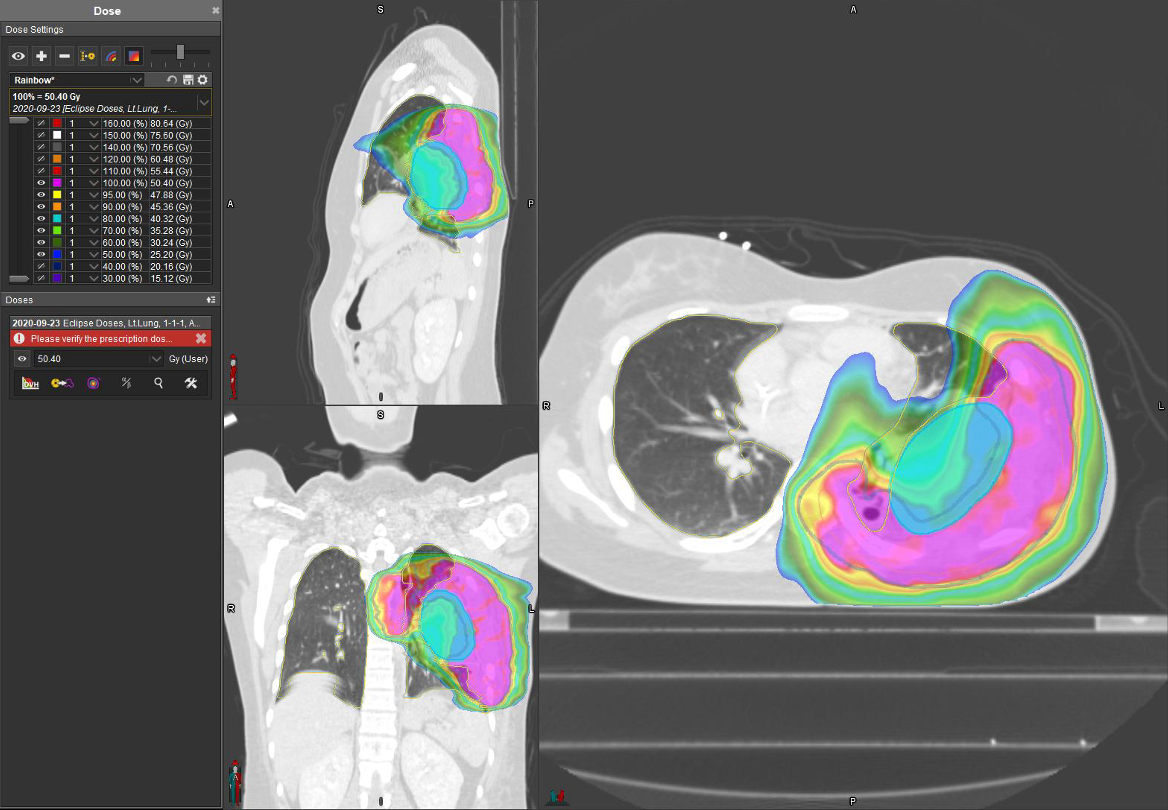

Supplement: Supplementary Figure 1 [file scr-11-01-25-0433-s001.tiff]
